# Social and behavioral research with end-users and healthcare providers into understanding perceptions of and reactions to a monthly oral contraceptive capsule in Bangladesh, Senegal and Zimbabwe

**DOI:** 10.3389/fgwh.2024.1433189

**Published:** 2024-12-11

**Authors:** Moushira El-Sahn, Rose Elliott, Trisha Wood Santos, Mona El-Sahn, Jeff Lucas

**Affiliations:** ^1^ Routes2Results, Sevenoaks, United Kingdom; ^2^ Routes2Results, Aberdeen, United Kingdom; ^3^ Routes2Results, London, United Kingdom; ^4^ Trisha Wood Santos Consulting, LLC, Seattle, WA, United States

**Keywords:** family planning, acceptability, preferences, contraceptives in development, Africa, Asia, women, attributes

## Abstract

**Introduction:**

Unintended pregnancy is a global public health challenge. Many contraceptive methods are available to end users, but non-use and discontinuation due to health concerns or side effects, particularly related to unpredictable or undesirable menstrual bleeding, are common. Oral contraceptive pills (OCPs) often have regular bleeding patterns compared to other hormonal contraceptives but require daily adherence. To address the issues of bleeding-related side effects and daily adherence, researchers are developing a monthly oral contraceptive (MOC) containing the same hormones as common OCPs. We studied women's and healthcare providers’ (HCPs’) perceptions of the MOC technology with the primary goal of providing feedback on product attributes to inform early design decisions.

**Methods:**

Our study consisted of a qualitative phase with in-person in-depth interviews (IDIs) with a total of 60 women of reproductive age in three regions in three countries (Bangladesh, Senegal, and Zimbabwe) and quantitative surveys, which took place after the qualitative phase, via face-to-face computer-assisted interviews of a total of 1,825 women in 17 regions in these three countries. We conducted 96 IDIs and 632 quantitative interviews with HCPs in one urban area per country.

**Results:**

Women's and HCPs’ perceptions of MOC product attributes were assessed and included a focus on potential menstrual changes and possible reasons for discontinuation. Overall, the most appealing attribute of an MOC was its 1-month duration. Women and HCPs alike preferred regular, monthly menstruation. Any unpredictable or irregular bleeding, including heavy bleeding or amenorrhea, would be a reason to discontinue an MOC if it were to have those attributes.

**Discussion:**

Overall, an MOC has a high and broad level of appeal amongst all the groups of women and HCPs who participated in the study and has a strong value proposition for important contraceptive needs including convenient duration, ease of use, discretion, and acceptable side effects. This appeal assumes that the experience of using an MOC would be very similar to that of daily OCPs except for dose timing. Future research on a hormonal MOC should include an exploration of women's tolerance and acceptability of potential side effects, particularly regarding bleeding, to validate its value proposition.

## Introduction

In 2019, there were approximately 111 million unintended pregnancies, of which 77% were among women with an unmet need for contraception or those who wanted to avoid pregnancy but were not using a modern method of contraception (1). Demographic and Health Surveys (DHSs) consistently report that side effects or health issues are stated reasons for the non-use of contraception (28% in Africa and 23% in Asia) (2). It is common to experience menstrual bleeding changes with the use of modern hormonal methods, indicating a potential correlation between non-use or discontinuation due to dissatisfaction and method-related side effects (3, 4). In fact, DHS data indicate that women who reported non-use due to side effects or health concerns were more likely to have previously used modern contraceptive methods such as implants, injectables, intrauterine devices (IUDs), or oral contraceptive pills (OCPs) (2). Furthermore, a recent review article on women's tolerance of contraceptive-induced menstrual bleeding changes (CIMBCs) reported that in over half of the studies, women perceived bleeding changes such as spotting or irregular or more frequent bleeding to be unacceptable (5).

Daily combined OCPs (containing estrogen and progestin) are a favored product among many users because of the regular monthly withdrawal bleed (which occurs when there is a change in hormones such as that during the hormone-free interval), but also due to their ease of use, wide availability, low cost, and high efficacy when used as directed. Yet, a lack of supply, forgetting to take the pill every day, or not taking it at the same time each day are possible reasons that OCPs have typical use failure rates three to eight times greater than in clinical trials (6–10).

To address failure rates linked to the daily adherence burden, once-a-month oral contraceptives have been developed. In the 1960s, scientists developed once-a-month contraceptive pills using quinestrol (estrogen) and norgestrel or levonorgestrel (progestogen) that were registered in China, but concerns have been raised about their safety given rates of side effects and hypertension associated with use (11). Mifepristone has also been studied for once-a-month use and has been shown to be efficacious and acceptable to women (12, 13). However, some women with varying beliefs, values, and experiences do not want to use a method that could interfere with the implantation of a fertilized egg (14). These insights highlight the importance of understanding different end user practices, views, and preferences when designing a new contraceptive product (14).

Historically, new contraceptives have been developed without much direct input from end users early in the design phase despite available research methodologies and expertise (15, 16). In more recent years, approaches that have been used in the commercial healthcare sector are being used to solicit user feedback and inform early product design, including for long-acting methods (16–21). Our study examines both end user and healthcare provider (HCP) preferences and attitudes in Bangladesh, Senegal, and Zimbabwe to provide key information for the early product design of a new monthly oral contraceptive (MOC). An MOC is a capsule containing a specially designed formulation that can deliver up to 3 weeks of contraceptive protection using a combination of two hormones found in daily OCPs, such as ethynyl estradiol and levonorgestrel, and thus would inhibit ovulation (22). A withdrawal bleed would follow the 3 weeks of contraceptive protection, mimicking monthly menstruation and signaling that the next monthly pill should be taken. Such a new MOC has the potential to provide women and adolescents seeking contraception with an option that addresses reasons for non-use, discontinuation, and method failure with fewer bleeding disruptions and better compliance.

Our study involved 1,935 women and 728 HCPs in Bangladesh, Senegal, and Zimbabwe with the primary objective of understanding the overall perception of an MOC, with a focus on product attributes. The countries in the study represent diverse OCP use rates (50% in Bangladesh, 19% in Senegal, and 57% in Zimbabwe) (23) to test the hypothesis that countries with strong OCP use rates are conducive environments into which to introduce an MOC. The secondary objective was to provide evidence for refining messaging and the value proposition based on a Consumer-Targeted Product Profile (CTPP). The tertiary objective was to understand the level of appeal and likelihood of trying an MOC.

## Methods

### Overview of project

Our study consisted of two phases: a qualitative phase followed by a quantitative phase. Respondents who participated in the qualitative component did not participate in the quantitative component. All the interviews were conducted at a place of the respondent's choosing, often in their home, or a space where they felt they could talk openly.

#### End users

For the qualitative phase, data were collected via in-person in-depth interviews (IDIs) with a total of *n* = 60 women in Bangladesh (Dhaka and Sreepur), Senegal (Dakar and Linguère), and Zimbabwe (Harare and Hurungwe). This sample was divided into *n* = 10 rural-based women in both Zimbabwe and Senegal, and *n* = 20 rural-based and *n* = 20 urban-based women in Bangladesh. A higher IDI sample size was implemented in Bangladesh due to the sensitivity of the topic and the consequent limitations of group discussions, preventing the possibility of qualitative focus groups, which were conducted in Senegal and Zimbabwe (*n* = 50, with 10 mini-workshops of *n* = 5 per workshop). As such, we have focused on shared methodologies in this paper. The qualitative work in Senegal and Zimbabwe was carried out prior to the work in Bangladesh. The respondents were asked to think about contraception broadly to situate them in the topic. Next, they reviewed all the provided stimuli (first a video, then a profile, and finally a prototype of the outer capsule). At each point, the respondents were asked to describe how they perceived the MOC, including benefits, challenges, concerns, and whether they would be willing to try it. Finally, respondents were asked about communication and how they would seek information on/tell others about an MOC. For the quantitative phase of the research, data were collected via face-to-face computer-assisted personal interviews (CAPIs) with a total of *n* = 1,825 women in Bangladesh (Dhaka *n* = 305, Chattogram *n* = 151, Rajshahi *n* = 80, and Khulna *n* = 81), Senegal (Dakar *n* = 206, Thiès *n* = 154, Saint-Louis *n* = 39, Kaolack *n* = 67, Ziguinchor *n* = 68, and Diourbel *n* = 67), and Zimbabwe (Harare *n* = 181, Manicaland *n* = 138, Mashonaland East *n* = 106, Bulawayo *n* = 87, Matabeleland *n* = 43, Mutare *n* = 38, and Masvingo *n* = 14).

#### HCPs

For the qualitative phase, data were collected via in-person IDIs with a total of *n* = 96 HCPs (*n* = 32 in Dhaka, Dakar, and Harare, respectively). For the quantitative phase, data were collected face-to-face via CAPIs with a total of *n* = 632 (Dhaka *n* = 79, Chattogram *n* = 51, Rajshahi *n* = 41, and Khulna *n* = 41), Senegal (Dakar *n* = 77, Thiès *n* = 57, Saint-Louis *n* = 13, Kaolack *n* = 22, Ziguinchor *n* = 22, and Diourbel *n* = 22) and Zimbabwe (Harare *n* = 62, Manicaland *n* = 46, Mashonaland East *n* = 36, Bulawayo *n* = 30, Matabeleland *n* = 15, Masvingo *n* = 9, and Mutare *n* = 8).

### Stimuli

The participants were exposed to the following stimuli:

Qualitative: A three-sentence description outlining the concept of the MOC without explaining its mechanism of action, images of the prototype (Supplementary Figure S1), initial CTPP, and two short informational videos (one animated lasting 54 s and the other a live-action demonstration lasting 53 s; both videos were silent, with an accompanying moderator script which was read to participants). The revised video, which incorporated important user learnings from the qualitative research which had been completed in Senegal and Zimbabwe (see quantitative stimuli below), was used in the qualitative research in Bangladesh, as this was carried out after the research in Senegal and Zimbabwe.

Quantitative: A three-sentence description outlining the concept of the MOC without explaining its mechanism of action (for end users only), images of the prototype (Supplementary Figure S1), revised CTPP following changes made in the post-qualitative phase, and a revised informational video lasting 1 min 48 s (a combination of aspects from the two videos in the qualitative phase, based on feedback from respondents).

Proprietary information on the mechanism of action has been redacted from the stimuli when reproduced here, and no information or results from the discussions concerning the video can be shared.

The three-sentence description is as follows: “This is a new contraceptive product that prevents unintended pregnancy when it's used correctly and consistently. It needs to be taken only 1 time per month, rather than daily like other oral contraceptives. It is intended to prevent pregnancy on a month-to-month basis”.

Reproduced in the Supplementary Material is the revised end user CTPP (Supplementary Figure S2), as used in the quantitative component of the study. This includes an informational section on the reproductive system and menstrual cycle which was not included in the CTPP shown to HCPs, which was otherwise the same apart from wording changes. All wording changes in the CTPP shown to providers are shown in Supplementary Figure S2.

### Population sample

#### End users

The target population was women of childbearing age (18–40 years for the qualitative phase, and 18–49 years for the quantitative phase) who self-reported being sexually active, were not currently pregnant, were not planning to conceive in the next year, and who were open to using contraceptives/family planning, in socio-economic classes (SEC) C and D. This research utilized the EquityTool which is a short, country-specific questionnaire to measure relative wealth (24). The C and D SEC strata were selected for this research as they encompass the broadest and largest section of the population and are critical target populations for contraceptive programs. Participants aged 18–40 years were selected as the age range for the qualitative sample as an upper limit of 40 years was considered to be able to provide an adequate representation of the older age range among childbearing women in the qualitative sample. For the quantitative sample, however, the 18–49 year age group was chosen in order for the quantitative results to be generalizable across the broader demographic of women of childbearing age in these locations (25, 26). Other demographic information was captured during the interviews; however, this did not determine eligibility. Aside from the eligibility criteria detailed above, there were no quotas for subdividing the sample, and the characteristics of the sample depended on the natural subject distribution following recruitment. The respondents who participated in the qualitative component did not participate in the quantitative component.

#### HCPs

HCP eligibility criteria included a set quota of provider types which comprised doctors, nurses, community health workers (CHWs), and pharmacists/medicine sellers. The provider types could operate within either a public or private setting and had to have been practicing for over 1 and be under 40 years old. The providers had to regularly (on a daily or weekly basis) provide counsel to women on family planning/use of contraceptives. Finally, providers were not eligible if they were working for any pharmaceutical company or manufacturer as a clinical trial investigator, consultant, researcher, or in any other capacity, and if they had participated in market research about family planning or contraceptive methods within the 6 months preceding the study.

The majority of our HCP sample were women (61.8% overall, 64.6% in Bangladesh, 62.0% in Senegal, and 58.7% in Zimbabwe). There were significantly more men in the doctor and pharmacist specialties (72.0% and 62.0% overall, respectively) and a significantly higher proportion of nurses and CHWs were women (80.0% and 86.3% overall, respectively).

### Country selection

The study selected countries based on the question of whether those with high OCP use would provide conducive environments for an MOC. This was done by contrasting two countries with high OCP usage (Bangladesh and Zimbabwe) with one with a mixed distribution of methods, including high proportions of long-acting reversible contraceptive (LARC) usage (Senegal) (23).

### Translations

All informed consent forms, stimuli, and research tools were translated into the main languages spoken in the areas where the fieldwork was conducted (Bengali in Bangladesh, French in Senegal, and Shona and Ndebele in Zimbabwe). The respondents were able to choose the language for the written materials and discussion and could switch during interviews if they preferred.

### Data collection

For the qualitative component, discussions were via face-to-face IDIs and lasted between 40 and 60 min. For data collection, the interviews were audio-recorded, transcribed, and, where necessary, translated prior to analysis. For the quantitative phase, women were surveyed via face-to-face CAPIs, which lasted between 30 and 35 min. For data collection, mobile phones/tablets with offline data storage capabilities were used and data were automatically uploaded when an internet connection was available.

The research teams, comprising female interviewers and recruiters and mixed-sex supervisors, were selected for their qualitative and quantitative competencies and background, and were briefed and trained. Pilot interviews were observed across all three countries, ensuring adherence to objectives, processes, and ethical considerations. Interviews were conducted by experienced interviewers in the local language or English, based on respondent preference.

Due to the COVID-19 pandemic, precautions were put in place to minimize risk. Inter-country travel was minimized; our study team briefed the in-country fieldwork teams remotely using video meeting technology. Fieldwork was only conducted when there were no government restrictions in place that would be contravened by carrying out this work. Guidelines were developed based on those provided for face-to-face interviewing by the European Society for Opinion and Market Research (ESOMAR) (25).

Provision was made for the use of alternative formats for completing the research such as telephone interviews or video interviews using programs such as Zoom or Skype if needed to comply with government restrictions; however, these provisions were not utilized as it was possible to conduct all research face to face.

### Data analysis

For the qualitative data, codebooks were developed iteratively following a review of transcripts by the core study team of four research directors, each with at least 5 years of qualitative analysis experience. This framework was used to code transcripts and identify key themes that emerged from the data. An iterative and systematic process of content and pattern analysis was carried out. The study team used the analytical categories developed as part of the coding framework to derive meaning from the various pieces of evidence to answer the research questions. The study analysis team met regularly to review codebook outputs, with a view to align/calibrate and/or resolve coding challenges which included a discussion and consensus building, revisiting the codebook, and a third-party review.

The closed-ended quantitative data were analyzed by initial base analysis including an examination of the data at a total respondent level, as well as by key groups such as marital status, across age groups, and urban vs. rural. In accordance with the conventional acceptance of statistical significance at a *P*-value of 0.05 or 5%, confidence intervals (CIs) were calculated at a confidence level of 95%. The tests used in the analysis included the paired/overlap *t*-test for means and the paired/overlap z-test for percentages. In general, if an observed result is statistically significant at a *P*-value of 0.05, then the null hypothesis should not fall within the 95% CI. Statistical significance (*P* ≤ 0.05) was observed when the differences in data from one country were not different from the findings in the other due to chance. There were, however, no significant differences between sample distinctions (demographics such as age, setting, contraceptive use, etc.) that were relevant to the objectives.

This study utilized a 5-point rating scale or Likert rating scale for some questions. When responding to a Likert rating scale question, the respondents specified their level of agreement or disagreement on a symmetric agree-disagree scale for a series of statements (27). Thus, the range captured the intensity of their feelings for a given item (27). Analysis of these questions is presented with data for each scale item and, in some cases, includes Top 2 Box scores, sometimes denoted as T2B (or “positive” scoring), and Bottom 2 Box (“negative” scoring) as a way of examining and presenting the responses to the Likert scale questions (28).

Open-ended data within the quantitative interviews were also analyzed. The process began with a review of the verbatim responses for each question. Common themes were identified, as well as factors associated with each theme. This represented a code frame. Each verbatim response was then analyzed and assigned an appropriate code.

## Results

Table 1 shows the demographic information concerning the respondents who took part in the research. In terms of quantitative end user location, the rural/urban (or peri-urban) distribution of respondents was as follows: 51.4% urban vs. 48.6% rural in Bangladesh; 69.0% urban vs. 31.0% rural in Senegal; 58.3% urban vs. 41.7% rural in Zimbabwe. The HCP sample for this study was *n* = 631, split fairly equally between four specialties: doctor (23.9% overall), nurse/family welfare visitor (FWV)/counselor (26.5%), CHW (24.6%), and pharmacist/medicine seller (25.0%). Table 2 shows the data across specialties and countries. Overall, the sample was split equally between those spending the majority of their time in either a public (50.4%, driven by Senegal and Zimbabwe with 56.8% and 55.3%, respectively) or private setting (49.6%, driven by Bangladesh with 60.8%), with the private setting being split further between hospitals/health clinics/facilities (27.9%) and pharmacies/medicine shops (21.7%).

**Table 1:** Demographic and motherhood-related data, self-reported.

	Total (*n* = 1,825)	BGD (*n* = 617)	SEN (*n* = 601)	ZWE (*n* = 607)
Age (%)				
18–23 years old	19.6	22.9	14.5^a^	21.3
24–30 years old	38.6	44.2	38.8	32.6^a^
31–35 years old	22.7	21.1	20.5	26.7^b^
36–48 years old	19.1	11.8	26.3^b^	19.4
Mean (years)	29.87	28.49	31.10	30.04
Relationship status (%)				
Married and living together^c^	77.5	96.4^b^	71.0	64.7
Single + BFs^d^	9.7	—	16.1	13.2
Married and not living together^e^	6.3	3.4	7.5	8.1
Not living together	1.8	—	0.2	5.3^b^
Living together	1.6	0.2	0.7	4.1^b^
Divorced	1.5	—	2.5	2.1
Widowed	0.4	—	0.3	0.8
Number of children (%)				
None	11.2	9.6	17.5^b^	6.8
1	25.1	28.7	20.8	25.7
2	33.2	44.2^b^	25.0	30.0
3	16.9	14.3	14.1	22.2^b^
4	7.8	2.8^a^	11.5	9.4
5	4.1	0.2^a^	3.7	1.2
6 or more	1.7	0.3^a^	3.7	4.8
Mean	1.95	1.75^a^	2.05	2.06
Education level (%)				
Primary + below	19.6	26.7	22.5	9.6
Junior secondary	35.3	39.9	18.8^a^	47.1^b^
Senior secondary	22.9	23.0^a^	15.8	29.8

Country names using Alpha-3 codes: BGD, Bangladesh; SEN, Senegal; ZWE, Zimbabwe.

^a^ Significantly lower than the two other countries.

^b^ Significantly higher than the two other countries.

^c^ Married and living with partner.

^d^ Boyfriend(s).

^e^ Married and not living with partner.

**Table 2:** Distribution of HCP respondent specialties across countries (number of respondents, *n* = 631).

	Total (*n* = 96)	BGD (*n* = 32)	SEN (*n* = 32)	ZWE (*n* = 32)	Total (*n* = 631)	BGD (*n* = 212)	SEN (*n* = 213)	ZWE (*n* = 206)
	Qualitative component (*n*)				Quantitative component (*n*)			
Doctor	24	8	8	8	151	51	50	50
Nurse/FWV/counselor	24	8	8	8	167	53	59	55
CHW	24	8	8	8	155	52	52	51
Pharmacist/medicine shop owner/medicine seller	24	8	8	8	158	56	52	50

Country names using Alpha-3 codes: BGD, Bangladesh; SEN, Senegal; ZWE, Zimbabwe.

The results described in this paper are the total of all three countries; country differences and other data cuts are expressed in the tables. Statistical significance is noted where a result from one country is significantly different from the other two (given as “significantly high” or “significantly low”). Percentages in the text and tables are given to one decimal place. Where predefined lists of reasons were provided in the quantitative questionnaire, these were informed by answers from the qualitative phase, with an “Other—specify” option also included. The results from the qualitative research are given to provide context for quantitative results.

## Context

### End user contraceptive awareness and use

*Quantitative:* The respondents were asked a variety of questions about their contraceptive awareness and use. The most frequently spontaneously recalled method was the daily OCP, mentioned by 89.8% of respondents. Next was the contraceptive injection, named by 57.0% of respondents. The third most named method was the contraceptive implant, mentioned by 50.4% of respondents. The results for all the methods mentioned are in Table 3. Note that no distinction was made between the combined pill (containing estrogen and progestogen) and the so-called mini pill (containing progestogen only).

**Table 3:** End user respondents’ unaided recollection of contraceptive methods.

	Total (*n* = 1,825) (%)	BGD (*n* = 617) (%)	SEN (*n* = 601) (%)	ZWE (*n* = 607) (%)
Oral birth control pill/“daily pill”	89.8	96.9^b^	83.7	88.5
Contraceptive injection/DMPA^c^/“shot”/“jab”	57.0	72.0	73.4	25.7
Contraceptive implant/“rod”/“strand”	50.4	21.9^a^	72.5^b^	57.3
Male condom	47.9	74.4^b^	18.0^a^	50.7
IUD/Copper T, “non-hormonal loop,” “copper loop,” or “coil”	37.4	24	27.8	60.5^b^
Levonorgestrel intrauterine system (LNG-IUS) or “hormonal loop”	18.6	—	4.3	51.7^b^
Female condom	13.9	—	1.8	39.9^b^
Oral emergency contraceptive pill/“emergency pill”	4.4	1.6^a^	3.8	7.7
Other methods	4.1	2.8	2.8	6.8^b^
Counting days/rhythm method	1.5	0.2	3.8^b^	0.5
Diaphragm/“cap”	0.8	1.0	0.5	1.0
Withdrawal	0.8	—	—	2.5
Contraceptive vaginal ring (CVR)	0.6	—	1.7	0.2
Spermicide/spermicidal jelly, foam, suppository, or film	0.5	—	0.2	1.3
Contraceptive patch	0.2	—	0.5	—
None	0.3	0.3	0.2	0.5

Country names using Alpha-3 codes: BGD, Bangladesh; SEN, Senegal; ZWE, Zimbabwe.

^a^ Significantly lower than the two other countries.

^b^ Significantly higher than the two other countries.

^c^ Depot medroxyprogesterone acetate.

The most reported aided awareness (respondents were shown a list of contraceptive methods displayed with a randomized image) was of the daily OCP, at 94.9%. The second most reported was the contraceptive injection, at 87.1% overall. Awareness of the male condom was the third most reported, at 77.4%. The results for all methods are listed in Table 4.

**Table 4:** End user respondents’ aided recollection of contraceptive methods.

Summary of top score	Total (*n* = 1,825) (%)	BGD (*n* = 617) (%)	SEN (*n* = 607) (%)	ZWE (*n* = 607) (%)
Oral contraceptive pill	94.9	96.8	91.7^a^	96.2
DMPA	87.1	90.4^b^	85.0	85.7
Male condom	77.4	93.5	45.6^a^	92.4
Contraceptive implant	69.3	48.8^a^	77.5	82.0
IUD	48.5	45.9	39.1^a^	60.5^b^
Oral emergency contraceptive pill	39.2	36.3	35.4	46.0^b^
Female condom	38.8	8.8^a^	22.1	86.0^b^
LNG-IUS	26.2	5.2^a^	26.5	47.3^b^
Contraceptive vaginal ring (CVR)	7.5	6.8	8.3	7.2
Diaphragm	7.1	4.4	5.3	11.7^b^
Counting days/rhythm method	5.8	1.6^a^	7.5	8.2
DMPA-SC^c^/bubble injection/Sayana Press	5.2	4.4	9.7^b^	1.5
Spermicide	4.6	3.1	3.0	7.7^b^
Contraceptive patch	3.3	3.1	4.3	2.6
Withdrawal	2.1	—	—	6.3
Other methods	0.9	0.3	1.3	1.2
None	0.3	0.2	0.5	0.3

^a^ Significantly lower than the two other countries.

^b^ Significantly higher than the two other countries.

^c^ Depot medroxyprogesterone acetate-subcutaneous.

The most reported method ever used was the daily OCP, at 73.5%. The next most used method was the male condom, used by 52.2%. The third most reported method was the contraceptive injection, at 36.5%. A minority of respondents, 3.1% overall (*n* = 57), specified that they had never used a contraceptive method. The results for all methods are listed in Table 5.

**Table 5:** Contraceptive methods ever used by the end user respondents.

	Total (*n* = 1,820) (%)	BGD (*n* = 606) (%)	SEN (*n* = 598) (%)	ZWE (*n* = 616) (%)
Oral contraceptive pill	73.5	87.8^b^	49.2^a^	82.8
Male condom	52.2	70.5^b^	23.7^a^	61.7
Contraceptive injection	36.5	37.3	37.1	35.0
Contraceptive implant	20.2	5.5^a^	31.1^b^	24.4
Oral emergency contraceptive pill	13.1	13.3	10.5	15.5
Female condom	6.0	1.1^a^	4.2	12.7^b^
IUD	4.9	3.1	7.0	4.6
Other methods^c^	3.4	1.1^a^	3.3^b^	5.8^b^
LNG-IUS	1.3	0.2^a^	2.2	1.7
DMPA-SC	0.8	—	2.5	—
CVR	0.7	0.3	1.5^b^	0.3
Diaphragm	0.4	—	0.8	0.5
Spermicide	0.3	—	0.5	0.3
Contraceptive patch	0.2	—	<0.1	—

Country names using Alpha-3 codes: BGD, Bangladesh; SEN, Senegal; ZWE, Zimbabwe.

^a^ Significantly lower than the two other countries.

^b^ Significantly higher than the two other countries.

^c^ Natural family planning, “the rhythm method,” or the “standard/calendar days”/“counting beads” method.

With regard to the methods currently used (respondents were able to specify more than one method), the most commonly used method was the daily OCP, at 48.2% overall. The next most used method was the contraceptive injection, at 16.7%. The third most used method was the contraceptive implant, at 13.9% overall, as shown in Table 6. A minority of respondents (11.1%) reported that they were not using any contraceptive method and 11.0% reported using the male condom. The proportion of respondents who reported usage of any other methods was between 0.1% and 2.7%.

**Table 6:** Contraceptives currently being used by the end user respondents.

	Total (*n* = 1,584) (%)	BGD (*n* = 545) (%)	SEN (*n* = 498) (%)	ZWE (*n* = 541) (%)
Oral contraceptive pill	48.2	58.7	30.3^a^	54
Contraceptive injection	16.7	14.7	23.3^b^	12.8
Contraceptive implant	13.9	1.8	24.7^b^	16.1
Male condom	11.0	19.4^b^	5.6	7.6
IUD	2.7	0.4	5.2^b^	2.8
Oral emergency contraceptive pill	1.2	1.5	1.0	1.1
Other methods^c^	0.9	0.7	1.6	0.4
LNG-IUS	0.8	—	1.6	0.9
Female condom	0.7	0.4	0.6	1.1
DMPA-SC	0.5	—	1.6	—
Diaphragm	0.3	—	0.6	0.2
Contraceptive vaginal ring (CVR)	0.3	—	1.0	—
Contraceptive patch	0.1	—	0.4	—
None	11.1	9.2	14.1	10.4
Mean	1.04	1.03	1.05	1.06

Country names using Alpha-3 codes: BGD, Bangladesh; SEN, Senegal; ZWE, Zimbabwe.

^a^ Significantly lower than the two other countries.

^b^ Significantly higher than the two other countries.

^c^ Natural family planning, “the rhythm method,” or the “standard/calendar days”/“counting beads” method.

Under a third of respondents (28.9%) stated that they had not visited a healthcare provider and discussed contraceptives in the past 12 months. Furthermore, 14.2% of the respondents reported one visit, 14.4% of the respondents reported two visits, and 12.2% of respondents reported three visits. The median number of visits reported was 2.0.

Most respondents (59.3%) specified they had received their most recent contraceptive from a healthcare facility. Pharmacies/medicine sellers were specified by 31.3%. A minority (9.4%) specified that they received their most recent contraceptive method from the community.

### HCP context

The median number of women of reproductive age (WRA) seen by the HCPs was 115 per month and the median number of women who consulted on contraceptive methods was 70 a month. The median number of women prescribed/offered a contraceptive method was 50. Providers in Bangladesh reported seeing more women than providers in the other two countries. This could be due to the nearly 10-fold larger population in Bangladesh (29) or the higher distribution of HCPs with 0.7 per 1,000 people in Bangladesh compared to 0.1 and 0.2 per 1,000 in Senegal and Zimbabwe, respectively (30).

To understand the age distribution of the women who consult HCPs regarding hormonal contraception, HCPs were asked to estimate the age distribution, in percentages, of the premenopausal women they see or consult in a typical month, specifically regarding prescription or hormone-based contraceptive methods or family planning. The mean percentage distribution across all three countries was as follows: <15 years old, 3.0%; 15–17 years, 9.2%; 18–25 years, 30.8%; 26–35 years, 31.9%; 36–45 years, 18.4%; >45 years, 6.6%.

Nearly all providers stated they had prescribed the daily OCP and the contraceptive injection (98.6% and 92.4% respectively). The next most recommended method was the contraceptive implant (76.1%) followed by the IUD (64.3%). Table 7 shows the full list of contraceptives ever provided.

**Table 7:** Prescription/hormonal contraceptives ever provided (or recommended in case of non-prescribers) by the HCP respondents.

**Contraceptive methods ever provided**	Total (*n* = 631) (%)	BGD (*n* = 212) (%)	SEN (*n* = 213) (%)	ZWE (*n* = 206) (%)
Contraceptive pill	98.6	99.5	99.1	97.1
Contraceptive injection	92.4	89.6	99.1	88.3
Contraceptive implant	76.1	62.3	88.7	77.2
IUD	64.3	59.9	88.3	44.2^a^
LNG-IUS	21.7	4.7^a^	39.9^b^	20.4
Another form of prescription contraception	21.4	14.2	15.0	35.4
Contraceptive vaginal ring (CVR)	10.6	2.8^a^	22.1	6.8

Country names using Alpha-3 codes: BGD, Bangladesh; SEN, Senegal; ZWE, Zimbabwe.

^a^ Significantly lower than the two other countries.

^b^ Significantly higher than the two other countries.

The method of contraception most provided in the previous 3 months was the daily OCP (mean percentage overall of 44.2%) followed by the contraceptive injection (25.8%) and the contraceptive implant (21.6%). Table 8 shows the full list of contraceptives provided in the previous 3 months.

**Table 8:** Prescription/hormonal contraceptives provided (or recommended in case of non-prescribers) in the 3 months preceding the study by the HCP respondents.

Contraceptive methods provided in the previous 3 months, summary of means (%)	Total (*n* = 631) (%)	BGD (*n* = 212) (%)	SEN (*n* = 213) (%)	ZWE (*n* = 206) (%)
Contraceptive pill	44.2	53.6	23.5^a^	56.2
Contraceptive injection	25.8	30.7	25.8	20.7
Contraceptive implant	21.6	13.5	29.5^b^	18.9
IUD	16.8	14.0	20.8	12.4
LNG-IUS	10.6	3.7^a^	11.8	9.8
Another form of prescription contraception	10.5	14.0	3.4^a^	12.2
Contraceptive vaginal ring (CVR)	6.9	7.5	6.2	9.0

Country names using Alpha-3 codes: BGD, Bangladesh; SEN, Senegal; ZWE, Zimbabwe.

^a^ Significantly lower than the two other countries.

^b^ Significantly higher than the two other countries.

HCPs were given a four-point scale regarding patients’ compliance with contraception: *poor*, *moderate*, *good*, and *unknown,* and asked to give a percentage for each, with the total amounting to 100%. Compliance was considered *good* by just over half of the HCPs (52.8%). Table 9 shows the mean percentages for patient compliance across all the levels.

**Table 9:** HCP perceptions of women's compliance with contraceptives’ instructions for use.

**Summary of means (%)**	Total (*n* = 631) (%)	BGD (*n* = 212) (%)	SEN (*n* = 213) (%)	ZWE (*n* = 206) (%)
Poor compliance	14.5	6.1^a^	23.3^b^	14.0
Moderate compliance	22.4	11.1^a^	28.9	27.3
Good compliance	52.8	75.4^b^	32.6^a^	50.5
Unknown compliance	10.3	7.4	15.2^b^	8.2

Country names using Alpha-3 codes: BGD, Bangladesh; SEN, Senegal; ZWE, Zimbabwe.

^a^ Significantly lower than the two other countries.

^b^ Significantly higher than the two other countries.

## Product attributes

### Familiarity with orally self-administered contraceptives

#### End users

*Qualitative:* The monthly capsule benefits from an established understanding and experience with some oral contraceptives. The monthly oral contraceptive was immediately compared with the daily OCP by respondents, and the vast majority found the critical difference (daily vs. monthly duration) to be the main driver behind the appeal.

### Monthly duration

#### End users

*Qualitative:* The once-a-month duration was a critical product attribute for the respondents, which, while perceived as having benefits in itself, also led to other perceived benefits for the end users. These benefits include ease of use and remembering while removing the burden of daily administration. Monthly oral contraception bridges the gap between daily and longer-term protection as well as offering those using LARCs an option to use without healthcare administration. The once-monthly duration was considered a favorable amount of time, making it easier to remember and self-administer, as was the lack of a need to visit an HCP or travel to a healthcare facility.

*Quantitative:* The respondents were asked two open-ended questions: (a) why they were interested in finding out more about the MOC having already watched a video on it, and (b) whether they were willing to try the MOC, having been exposed to the CTPP. The majority of women who were interested in finding out more and who were willing to try the MOC stated that it was because of its monthly duration and the ease of use associated with a monthly duration, at 50.8% and 49.4% respectively. Other reasons for interest or willingness to try were mentioned; however, all were mentioned by a comparatively low proportion of the respondents (under 10%).

The respondents were asked how important a number of features of a contraceptive method were to them, using a 5-point Likert rating scale from *Not at all important* to *Very important*. The list of features and data for all three countries can be found in Table 10. The most important feature selected by 89.6% of respondents within the T2B was “It is easy to remember when to take it or renew it.”

**Table 10:** Importance of features of a contraceptive method for the end user respondents.

Top 2 Box scores, 4 + 5 out of 5	Total (*n* = 1,825) (%)	BGD (*n* = 617) (%)	SEN (*n* = 601) (%)	ZWE (*n* = 607) (%)
It is easy to remember when to take it or renew it	89.6	92.9	86.2	89.8
It is discreet. No one knows that I am taking/using it	83.5	89.3	91.7	69.4^a^
It does not cause irregular periods while using it	83.2	87.4	77.7^a^	84.5
When side effects occur, they are not severe enough to disrupt my daily life	82.7	86.7	76.0^a^	85.3
It allows me to become pregnant within 6 months of discontinuing it	82.3	93.5^b^	74.7	78.4
It does not cause my periods to stop while using it	82.0	87.5^b^	79.0	79.2
It does not cause side effects such as headaches or weight gain	81.4	85.7	76.5^a^	81.9
It does not have to be taken every day	78.8	83.1	79.9	73.5^a^
It protects against disease/infection	78.1	88.5^b^	71.5	74.0

Country names using Alpha-3 codes: BGD, Bangladesh; SEN, Senegal; ZWE, Zimbabwe.

^a^ Significantly lower than the two other countries.

^b^ Significantly higher than the two other countries.

Respondents were later shown the same list and asked how much they agreed that the MOC would fulfill the feature requirement on a 5-point rating scale from *Completely disagree* to *Completely agree*. There was a high level of agreement by the majority of the respondents on all the tested features, with “It is easy to remember when to take it or renew it” scoring the highest T2B. Table 11 presents the respondents’ agreement levels by country.

**Table 11:** How much the end user respondents agree that the MOC fulfills specified features of a contraceptive method.

Top 2 Box scores, 4 + 5 out of 5	Total (*n* = 1,825) (%)	BGD (*n* = 617) (%)	SEN (*n* = 601) (%)	ZWE (*n* = 607) (%)
It is easy to remember when to take it or renew it	88.7	95.5^b^	81.4^a^	89.1
It is discreet. No one knows that I am taking/using it	86.1	88.0	83.4	87.0
It will allow me to become pregnant within 6 months of discontinuing it	71.1	84.4^b^	58.4^a^	70.2
It may reduce period cramps and may reduce the symptoms women can experience pre-menstruation (PMS) such as bloating, headaches, and changes in mood	60.5	67.7^b^	55.2	58.3
It may clear up acne	52.3	61.8^b^	50.2	44.8
It may cause my period to occur less than once a month	49.3	51.4	46.9	49.4
It may cause spotting or bleeding between periods	39.0	46.8^b^	31.6^a^	38.4
It may cause my periods to stop while using it	37.0	40.0	33.6	37.2

Country names using Alpha-3 codes: BGD, Bangladesh; SEN, Senegal; ZWE, Zimbabwe.

^a^ Significantly lower than the two other countries.

^b^ Significantly higher than the two other countries.

### End user discontinuation of the daily OCP

*Quantitative:* The respondents who had reported ever using the daily OCP but who were not currently using it were asked to select the reasons why from a predefined list (based on results from the qualitative phase; an “Other—specify” option was also provided). The sample size for this question was *n* = 606, as opposed to the total sample size of *n* = 1,825. The most selected reason for discontinuation was “It was hard to remember to take it every day/not convenient” (33.3%). The second most common reason, driven by Bangladesh, was “I experienced headaches/migraines from it” (24.8%). The third most mentioned reason was “It upset my stomach/caused nausea” (22.4%). Table 12 shows all the reasons mentioned by the respondents.

**Table 12:** The reasons given by the end user respondents for discontinuing the oral contraceptive pill.

	Total (*n* = 606) (%)	BGD (*n* = 235) (%)	SEN (*n* = 156) (%)	ZWE (*n* = 215) (%)
It was hard to remember to take it every day/not convenient	33.3	22.1^a^	39.1	41.4
I experienced headaches/migraines from it	24.8	40.0^b^	12.2	17.2
It upset my stomach/caused nausea	22.4	43.4^b^	5.8	11.6
I gained weight from it	16.7	21.3	16.0	12.1
I didn’t like it	15.2	16.6	12.8	15.3
I didn’t like that my periods became irregular while I was using it	8.3	4.7	7.1	13.0
It wasn’t very effective at preventing pregnancy	4.8	2.6	3.8	7.9
It was difficult to find/not available	4.5	0.4	<0.1	12.1
I haven’t needed contraception since I stopped taking the contraception pill	4.1	0.4	6.4	6.5
My friends suggested I try something different	3.8	1.3	1.3	8.4
My doctor/nurse suggested I try something different	3.6	2.6	5.1	3.7
My partner did not like it	3.5	2.6	4.5	3.7
I had difficulty swallowing it	3.1	5.5	<0.1	2.8
I heard bad/worrying things about it	3.0	2.1	2.6	4.2
I was worried it may prevent me from being able to have a baby in the future if I decide I want one/another one	2.8	2.6	5.8	0.9
I had to travel long distances to get it	2.1	0.9	<0.1	5.1
I didn’t like that it reduced my monthly bleeding	1.7	2.1	0.6	1.9
I didn’t like that it stopped my monthly bleeding	1.7	0.9	3.8	0.9
It was too expensive	1.5	1.3	0.6	2.3
I didn’t like still getting my periods while using it	1.5	1.3	0.6	2.3
It wasn’t very effective at stopping the spread of disease/infections	1.0	—	1.9	1.4

Country names using Alpha-3 codes: BGD, Bangladesh; SEN, Senegal; ZWE, Zimbabwe.

^a^ Significantly lower than the two other countries.

^b^ Significantly higher than the two other countries.

#### HCPs

*Quantitative:* HCPs were asked how appealing they found the MOC, using a 5-point Likert scale ranging from *Very unappealing* to *Very appealing*, and those who rated their appeal in the T2B were asked to provide a reason. The providers reported the once-a-month duration as the main appeal (42.5%). The remaining reasons among a minority of providers (between 11.7% and 15.4%) included: *Easy to use*, *Easy to remember*, and *Easy to swallow*. Providers were also asked why they thought women would find an MOC appealing, and the leading reason was *Once-a-month duration* (42.9%). The second and third reasons were *Easy to remember* and *Convenient* (23.9% and 11.1% respectively).

### Capsule size

#### End users

*Qualitative:* The end users were asked their views on the size of the capsule and whether they thought they would be able to swallow it easily. The respondents in all three countries remarked on the MOC capsule being large, with this being something most respondents in Senegal would like to change, but to a lesser extent in Zimbabwe and Bangladesh, where fewer than half (Zimbabwe) and a smaller minority (Bangladesh) of respondents said they would change the size. However, almost all respondents indicated that they believed they would be able to swallow the capsule, with the two main reasons being that it could be swallowed easily with water and that, being a capsule, it would have a slippery coating.

*Quantitative:* Having seen the video and CTPP, the respondents were asked to state how easy they thought the capsule would be to swallow. Ease of administration was evaluated on a 5-point Likert scale ranging from *Very difficult* to *Very easy*. The vast majority of participants felt that the capsule would be either somewhat or very easy to swallow (86.4% within the T2B). The mean score overall was 4.36.

#### HCPs

*Quantitative:* The confidence of the HCPs in a woman's ability to swallow the capsule was evaluated on a 5-point Likert scale ranging from *Not at all confident* to *Very confident.* Nearly all (91.3%) respondents reported being *Confident* or *Very confident*.

## Menstruation experience

### Predictability

#### End users

*Quantitative:* The respondents who had ever used a contraceptive method were asked to report whether they had experienced any of the following contraceptive-related bleeding patterns: *Spotting in between periods* (25.9%), *Prolonged bleeding/bleeding for longer than usual* (21.7%), *Bleeding occurring less frequently than once a month* (31.4%), *Bleeding occurring more frequently than once a month* (23.2%), or *None of the above* (43.5%)*.* In total, 61% of the sample reported currently using the daily OCP (see Table 6), meaning that withdrawal bleeding was occurring rather than menstruation.

### Unscheduled bleeding patterns

*Quantitative:* The respondents who had ever used a hormonal contraceptive method (*n* = 1,663) were asked to report whether they had experienced any of the following contraceptive-related bleeding patterns: *Spotting in between periods*, *Prolonged bleeding/bleeding for longer than usual*, *Bleeding occurring less frequently than once a month*, *Bleeding occurring more frequently than once a month*, or *None of the above.* Under half, albeit the biggest single proportion (42.6%), reported that they had not experienced any of the unscheduled bleeding patterns. A third (32.1%) reported bleeding occurring less frequently, driven by Bangladesh (44.3%, significantly high). Approximately a quarter reported experiencing the remaining unscheduled bleeding patterns, with significantly more respondents in Zimbabwe reporting experiencing these patterns. All the data are presented in Table 13.

**Table 13:** Unscheduled bleeding patterns experienced by the end user respondents while using a contraceptive.

	Total (*n* = 1,663) (%)	BGD (*n* = 582) (%)	SEN (*n* = 511) (%)	ZWE (*n* = 570) (%)
Bleeding occurring less frequently than once a month	32.1	43.3^b^	24.7	26.1
Spotting in between periods	26.7	19.6^a^	26.2	34.4^b^
Bleeding occurring more frequently than once a month	23.6	22.2	18.6	29.6^b^
Prolonged bleeding/bleeding for longer than usual	21.9	17.4	17.4	30.7^b^
None of the above	42.6	41.2	47.0	40.0

Country names using Alpha-3 codes: Bangladesh (BGD), Senegal (SEN), and Zimbabwe (ZWE).

^a^ Significantly lower than the two other countries.

^b^ Significantly higher than the two other countries.

Those who had experienced unscheduled bleeding patterns or period stoppage (*n* = 1,204) were asked to rate how concerned they felt during various scenarios on a 3-point Likert scale ranging from *Not at all concerned* to *Very concerned*, with an option for *I don’t remember*. Overall, the mean scores were high across all bleeding patterns/stoppage, indicating that they were very concerned. The mean ranged from 2.13 out of 3.00 for *Bleeding occurring less frequently than once a month* to 2.54 out of 3.00 for *Prolonged bleeding/bleeding longer than usual*. Table 14 shows the data across all three countries and score levels.

**Table 14:** Levels of concern felt by the end user participants due to experiencing unscheduled bleeding patterns while using a contraceptive.

	Total (*n* = 1,204) (%)	BGD (*n* = 396) (%)	SEN (*n* = 391) (%)	ZWE (*n* = 417) (%)
Spotting in between periods (*n*)	470	120	143	207
Very concerned (3)	41.3	55.0	15.4^a^	51.2
Somewhat concerned (2)	29.6	31.7	37.1	23.2
Not at all concerned (1)	25.1	12.5	39.9^b^	22.2
I don’t remember	4.0	0.8	7.7	3.4
Mean	2.17	2.43	1.73^a^	2.30
Prolonged bleeding/bleeding for longer than usual (*n*)	393	109	99	185
Very concerned (3)	65.1	59.6	52.5	75.1^b^
Somewhat concerned (2)	17.3	22.9	23.2	10.8
Not at all concerned (1)	13.2	16.5	14.1	10.8
I don’t remember	4.3	0.9	10.1	3.2
Mean	2.54	2.44	2.43	2.66^b^
Bleeding occurring less frequently than once a month (*n*)	566	270	139	157
Very concerned (3)	37.8	38.1	27.3^a^	46.5
Somewhat concerned (2)	31.8	40.0	33.8	15.9
Not at all concerned (1)	25.6	19.6^a^	33.1	29.3
I don’t remember	4.8	2.2	5.8	8.3
Mean	2.13	2.19	1.94^a^	2.19
Bleeding occurring more frequently than once a month (*n*)	423	137	110	176
Very concerned (3)	61.0	58.4	40.0^a^	76.1
Somewhat concerned (2)	19.1	25.5	28.2	8.5^a^
Not at all concerned (1)	13.5	13.9	18.2	10.2
I don’t remember	6.4	2.2	13.6	5.1
Mean	2.51	2.46	2.25^a^	2.69
Period completely stopping (*n*)	550	134	222	194
Very concerned (3)	53.6	72.4^b^	43.2	52.6
Somewhat concerned (2)	16.5	10.4	25.2^b^	10.8
Not at all concerned (1)	23.8	15.7	23.4	29.9
I don’t remember	6.0	1.5	8.1	6.7
Mean	2.32	2.58	2.22	2.24

Country names using Alpha-3 codes: BGD, Bangladesh; SEN, Senegal; ZWE, Zimbabwe.

^a^ Significantly lower than the two other countries.

^b^ Significantly higher than the two other countries.

### Amenorrhea

*Quantitative:* Of those respondents who had ever used a contraceptive method (*n* = 1,763), 69.6% said their periods did not completely stop whilst using a method.

## Menstruation preferences

### Amenorrhea vs. regular periods

#### End users

*Qualitative:* Amenorrhea was considered unacceptable by almost all respondents. There was a high importance placed upon periods as part of a woman's life, particularly in Senegal. Having a period was considered important because it was perceived to be healthy, normal, and to have a cleansing effect on the body; these views were consistent across all three countries. Furthermore, periods were widely viewed as a reassuring indication of not being pregnant. A small minority of respondents in Bangladesh and Zimbabwe indicated that amenorrhea would be acceptable, but for different reasons: as long as they were informed of this and it was explained that it was a side effect (Bangladesh), or it did not have an impact on fertility (Zimbabwe).

*Quantitative:* When asked if they prefer a product that allows for a regular period each month or a product that causes periods to stop occurring while using it, the majority preferred a regular monthly period (89.3% overall), with a small minority preferring a product that causes amenorrhea (7.0%).

#### HCPs

*Qualitative:* Overall, HCPs thought that women would largely prefer to have regular periods, although some thought that some women would accept amenorrhea; very few thought that it would be preferred. While the preference for periods was largely consistent across countries, this view was expressed by proportionally more respondents in Senegal, followed by Zimbabwe and Bangladesh. In Zimbabwe, multiple HCPs stated that women believed that periods cleanse the body of bad blood or dirt, a view which was expressed by only one HCP in each of the other countries. A significant reason given for women wanting regular periods was that they provide reassurance that one is not pregnant; HCPs also expressed the view that women believed that periods showed that they were healthy, or that conversely, the absence of periods could mean either that they were sick or that the contraceptive was harming them in some way. Of the HCPs who stated that amenorrhea would be acceptable, or acceptable for some, the importance of education was highlighted; it was believed that amenorrhea could be accepted only if the women were made aware that it was a side effect of the contraceptive and was not harmful, and that some women accept this now with other contraceptives (this was particularly mentioned in Bangladesh). In Zimbabwe, expense was mentioned as a reason to prefer amenorrhea, given the cost of sanitary products.

### Timing of monthly oral contraceptive administration

#### End users

*Quantitative:* The respondents were asked to state their preference regarding three options for when to take an MOC. Option 1: *You take the next capsule after 28 days each month (regardless of when you have your period)* (38.1%), option 2: *You take the next capsule once your period starts* (24.2%), and option 3: *You take the next capsule within 5 days of when your period starts each month* (24.6%). The respondents were also able to state whether they had no preference (13.0%).

### Side effects and discontinuation

*Qualitative:* Perceived abnormality, both in terms of menstruation and from other side effects, was thought to be disruptive to the end users’ lives in terms of peace of mind, relationships, time, and money. While overall women indicated a preference to experience non-disrupted periods, there was a general sense that they may be willing to trade disruption for an MOC's perceived benefits. Disrupted periods were considered better than amenorrhea, and lighter bleeding was typically not considered to be disruptive. Uncertainty was disruptive. Overall, the respondents across all three countries indicated that they would prefer predictability (a monthly period) and would discontinue if they experienced destabilizing side effects such as heavy bleeding.

*Quantitative*: A 5-point Likert scale was used, ranging from *Definitely would discontinue* to *Definitely would not discontinue,* to assess the likelihood of end users discontinuing MOC use if they were to encounter a specified list of menstruation-related side effects. The respondents indicated whether the specified side effects would likely prompt discontinuation by selecting either option 1 (*Definitely would discontinue*) or 2 (*Probably would discontinue*). The side effects listed were heavy bleeding, increased menstrual pain above the normal level, irregular periods, missing a period, shorter but regular periods, and lighter periods.

Heavy bleeding was the side effect that prompted the highest stated likelihood to discontinue (73.5%). Increased menstrual pain had a moderately high potential to prompt discontinuation (61.7%). Irregular periods and missing a period were indicated as somewhat likely to prompt discontinuation (59.3% and 55.0%, respectively).

In terms of side effects that would be less likely to prompt discontinuation, shorter but regular periods (29.1%) and lighter periods were indicated (34.3%).

#### HCPs

*Qualitative:* Across all three countries, HCPs specified that bleeding-related side effects as a whole were the main reason for contraceptive discontinuation. Nausea/vomiting was the second most given reason, again across all three countries; other frequently mentioned reasons were weight gain, headache, and, by a moderate number of HCPs, negative impact on libido.

*Quantitative:* While HCPs were not specifically asked about reasons for discontinuation, they were asked how frequently women express concerns about a specified list of aspects of contraceptives, including the following bleeding-related side effects: *Causing episodic bleeding or spotting*, *Causing missed menses*, *Causing amenorrhea*, and *Causing prolonged bleeding*. All of these were indicated by over half, and in many cases over 70% of the respondents, as being raised either very or somewhat frequently (Table 15).

**Table 15:** Menstrual side effects which women were reported to express concern about either very frequently or frequently by the HCP respondents.

Top 2 Box scores, 4 + 5 out of 5	Total (*n* = 631) (%)	BGD (*n* = 212) (%)	SEN (*n* = 213) (%)	ZWE (*n* = 206) (%)
Causing episodic bleeding or spotting	77.7	76.9	76.1	80.1
Causing missed menses	76.1	71.7	85.9^a^	70.4
Causing amenorrhea (no bleeding at all)	61.6	55.7	76.1^a^	52.9
Causing prolonged bleeding	65.5	67.0	53.5^b^	76.2

Country names using Alpha-3 codes: BGD, Bangladesh; SEN, Senegal; ZWE, Zimbabwe.

^a^ Significantly higher than the two other countries.

^b^ Significantly lower than the two other countries.

### Access

*Qualitative:* The end users indicated a general preference for obtaining the MOC at a healthcare facility (including family planning clinics) or pharmacy/medicine seller (particularly in Bangladesh) and taking it at home. There were country differences in challenges accessing healthcare facilities. In Bangladesh, long working hours and busy lives caused time limitations on visiting facilities. In Senegal, transport costs were mentioned. In the rural areas of Zimbabwe, irregular income due to seasonal variation, such as selling a tobacco harvest or livestock, led to a need to plan when to purchase items, as well as to factor in the high travel and time costs involved in visiting facilities.

*Quantitative:* The most popular access locations (from a pre-provided list) for end users to access an MOC were a clinic/family planning clinic (34.0%) and the pharmacy/medicine seller (33.0%). In terms of where respondents would like to take the MOC, most respondents selected at home (59.2%), followed by a health clinic/family planning clinic (20.0%) and the doctor's office (5.8%).

### Likelihood to try

#### End users

*Qualitative:* Across all three countries, the end user respondents in the qualitative part of the study demonstrated a high level of interest in trying the MOC. Reasons for this included convenience because of reduced pill burden, discretion, and perceived affordability benefits related to reduced financial and time commitments surrounding travel to healthcare facilities.

*Quantitative:* The end users were asked to provide usage intent regarding the MOC, having learned about it from an HCP and if it were available to them, on a 5-point Likert scale, ranging from *Definitely do not want to use* to *Definitely want to use*. Intent to use was defined as having selected option 4 or 5, T2B. The majority of the end users showed intent to use (83.0%).

#### HCPs

*Qualitative:* HCPs were asked whether they would recommend the MOC, whether they would recommend it to a particular patient group, and whether there were any patients for whom they would not recommend it. The vast majority of HCPs indicated they would recommend it, for similar reasons as those the end users outlined regarding their willingness to try: increased convenience and a perceived reduction in forgetting to take the pill. There were no identifiable patient groups for whom it was felt to be particularly suitable, with HCPs across all three countries stating that its appeal would be broad or specifying varied ranges of groups. In terms of patients for whom it would not be recommended, this was governed by the typical precautions regarding the risks of combined hormonal contraceptives in relation to medical conditions such as hypertension, or circumstances such as being over the age of 35 and a smoker.

*Quantitative:* The end users were asked to evaluate the appeal of an MOC on a 5-point Likert scale, ranging from *Very unappealing* to *Very appealing.* The majority of the end users perceived the MOC to be either somewhat or very appealing (86.5%).

HCPs were also asked about the likelihood of them providing the MOC to premenopausal women on a 5-point Likert scale. The majority of HCPs reported a high likelihood to provide an MOC (*Would definitely provide* or *Likely to provide*, 78.4%).

### Multipurpose prevention technology (MPT)—combined protection from HIV and unintended pregnancy

#### End users

*Qualitative:* The end users were asked about their views on combining contraceptive protection with HIV prevention within the MOC capsule. Across all three countries, the views on this idea were generally positive, on the grounds that it would provide important protection and that it would have the benefit of having two functions within one product. The respondents specified a variety of women for whom they felt this would be suitable, including those who were not able to trust their partner's fidelity and sex workers. Potential stigma was noted as a downside in Zimbabwe, in that respondents felt that male partners may feel that their female partner is HIV positive, is being unfaithful to them, or implying that they are unfaithful.

*Quantitative:* The end users were asked whether a dual-prevention product such as an MOC providing protection against both pregnancy and HIV would be of interest, to which nearly all answered *Yes* (94.4%).

#### HCPs

*Qualitative:* The HCPs were asked about combining contraceptive protection with both HIV prevention and antiretroviral therapy (ART). The views were mixed but positive overall. The reasons for thinking that dual prevention was a good idea and would be appealing to women included the idea of one medication with two functions, discretion, and a reduction in HIV incident cases. The reasons for negative opinions included the view that the MOC should only be for one purpose and that it could cause confusion, and that it could be seen as promoting unprotected sexual intercourse. Some HCPs thought it would be useful for sex workers and other at-risk populations. In terms of combining pregnancy prevention with HIV treatment, the views were mainly positive, with a strong indication that there would be high demand for this. Pill burden was highlighted here, with HCPs feeling that monthly pills would be better than daily. In terms of country differences, in Senegal, there was more discussion of HIV as taboo, and a small minority of HCPs in Senegal were not aware of the current existence of preventative drugs for HIV; some felt that the combination of the two types of protection was not necessary. The views in Bangladesh were generally positive, with HCPs confident that there would be demand for such a product. In Senegal and Zimbabwe, challenges in dispensing were highlighted with regard to combining contraceptive protection with antiretroviral (ARV) treatment, as patients may not wish to reveal their HIV status.

*Quantitative:* The HCPs were asked whether they would be interested in providing or recommending a dual-prevention product such as an MOC providing protection against both pregnancy and HIV, to which virtually all answered *Yes* (94.3%).

## Discussion

This paper describes user perceptions of an MOC in Bangladesh, Senegal, and Zimbabwe with a focus on preferred product attributes, appropriate messaging, and the value proposition, to inform further product development and the eventual introduction and promotion of this contraception method. An MOC has broad and high appeal for the women and HCPs in our study given its duration, familiar form, predictable monthly menstruation, discretion, convenience, and acceptable side effects. Overall, an MOC has higher appeal among study participants from Bangladesh than Senegal or Zimbabwe with a lower proportion of participants from Senegal favoring key attributes such as duration and pill size and expressing willingness to try an MOC. This may be due to the lower overall use of OCPs in Senegal than in Bangladesh or Zimbabwe and could reflect a preference for longer-acting methods such as implants. These findings confirm our hypothesis that countries with high OCP use are strong candidates for the initial introduction of an MOC as end user preferences align with the developed CTPP and support the value proposition of the method, i.e., its convenient duration, ease of use, discretion, and favorable side effect profile.

Over 60% of the participants in our study reported current use of a daily OCP and it was the most reported method ever used among the participants overall. A daily OCP offers benefits to women and adolescents as it is user-controlled, discreet, affordable, and can be accessed at many locations. Despite high current use of the OCP, approximately one-third of study participants had used the OCP in the past but discontinued. The reasons driving their discontinuation of the OCP were inconvenience (daily pill burden and the travel associated with it), difficulty remembering to take the pill every day, and the side effects experienced such as headache. These responses align with other studies that have found the reasons for the discontinuation of daily OCPs being linked to accessing and using the pills correctly (31) and dissatisfaction due to side effects (32).

Our study examined end user and HCP preferences for different attributes of the MOC including duration of pregnancy protection, capsule size, predictability of menstruation, timing of administration, side effects, and access. The once-a-month duration was the most appealing attribute for end users and HCPs alike because it is easy to use and remember, removing the need for daily administration and lending itself to higher typical use efficacy rates than daily OCPs (6–10). The monthly duration was also the primary reason for the study participants expressing an interest in learning more about and trying the MOC, which aligns with what participants stated as the most important feature of any contraceptive: being easy to remember to take or renew.

Our study confirms that the proposed MOC capsule size was acceptable and was perceived as very or somewhat easy to swallow. Furthermore, the study confirms that taking the pill every 28 days, or approximately every 1 month, was the preferred option regardless of when a woman's period starts. The study participants were confident in being able to remember a monthly dosing schedule and a monthly withdrawal bleed could serve as a reminder to take the next dose. Preferences regarding these product attributes were aligned across the three countries.

The women's feedback on the MOC stressed the importance of minimizing side effects, especially unpredictable ones that may interfere with their daily lives. Nearly 90% of women in our study indicated that they want monthly menstruation for reasons including it being healthy or normal, cleansing the body, and as an assurance that a woman is not pregnant. The women in our study from Bangladesh were more likely to discontinue the MOC due to bleeding changes than the women from Senegal and Zimbabwe where there were more women who would accept certain bleeding changes. For example, less than half of the study participants from Zimbabwe were likely to discontinue the MOC if it caused irregular periods, a missed period, shorter periods, or lighter periods. This may be due to greater use and experience with hormonal methods that result in bleeding changes in Senegal and Zimbabwe than in Bangladesh. Unanimously, the women indicated that other changes in bleeding patterns such as heavy bleeding, skipping or irregular menstruation, amenorrhea, or increased pain would prompt discontinuation of the MOC. These findings support that one of the main benefits of the MOC is having a regular withdrawal bleed, which contributes to greater continuation, and aligns with what is known about women's dislike and discontinuation of existing contraceptive methods (3–5).

The HCPs raised additional questions about the MOC that will be important to explore as the method is further developed and clinical studies are undertaken. The HCPs wanted to know more about the product's safety and efficacy including interactions between the MOC and other medications. In Senegal, the HCPs expressed concern about what to do if a client found the side effects intolerable and could not discontinue the method, and, in Zimbabwe, the HCPs were concerned about safety linked to overdosing or for cardiovascular disease. The HCPs were also concerned that the MOC could displace condom use and protection against sexually transmitted infections (STIs), particularly among those using condoms for contraception. The HCPs indicated that women who are interested in the MOC will want to know its effectiveness, side effects including amenorrhea, and their return to natural fertility after use. When planning the introduction of the MOC, it will be important to design and test appropriate messaging and information on the MOC. This will include how it is used, possible side effects, the importance of condom use to protect against STIs, and contraindications for HCPs and the women themselves.

An MOC has great potential for wide availability and self-use, alleviating the time and resources required from end users to access contraception and HCPs to provide family planning. Preferences regarding where to access the MOC vary by geography. The study participants from Bangladesh were split in their preference for the pharmacy/drug shop/medicine seller and health/family planning clinics. The participants from Senegal and Zimbabwe preferred a healthcare facility or clinic. Women primarily want to take the pill in the privacy of their own homes. Upon the initial introduction of the MOC, it is important to have broad availability to increase awareness and ensure women have the knowledge, skills, and confidence to use it on their own in the future.

### Additional research

Conducting this study early in the development of the MOC is important to align the design with end user preferences and maximize the potential for uptake once the product is available. More qualitative research would help uncover gaps in understanding the reasons driving certain preferences such as why women would prefer to access the product from a healthcare provider or facility and what drives that motivation, or, in the case of Senegal, why there was a lower percentage of women interested in the MOC compared to the other two countries and what underlies their concerns or hesitancy. Other areas to test with users include instructions for use and packaging appeal as the MOC is further developed. Developing and testing messaging to the end users and appropriate counseling messaging for HCPs will be important, particularly regarding questions related to product safety and side effects.

It is important to conduct actual use studies to understand the user's perspective on side effects and their impact on uptake and continuation. The study participants disliked changes in their menstrual bleeding, but also other side effects such as weight gain and headaches. However, the extent to which these would influence uptake and continuation is unknown.

Product launch planning will be important as the MOC is developed. Data on acceptable pricing for procurers and end users are important, as well as segmentation and demand forecasting to support decision-making on funding and manufacturing needs. Demand forecasts should also take into consideration all current contraceptive options on the market to understand possible shifts in the use of other methods when the MOC is introduced. Furthermore, research to inform countries’ policies that impact the accessibility and availability of the method, particularly regarding private sector pharmacy or drug shop access, may be necessary.

### Limitations

This study explores end user preferences for and HCP perspectives on a novel monthly contraceptive but has limitations. The study was designed to understand the stated preferences for the MOC and did not explore the preferences for the MOC vs. other methods. Many women in our study were current users of OCPs, which could cause significant shifts in demand from OCPs to the MOC, resulting in a limited impact in reducing unintended pregnancies. Understanding continuation rates with the actual use of the MOC will be important to better define its potential impact in meeting women's needs for family planning.

We do not recommend drawing broad conclusions across and between different country populations. The qualitative portion of the study is indicative in nature, and further, qualitative samples often fluctuate as not all questions are asked or answered. The scope of our research did not include data for a segmentation, a forecast, or modeling of product pricing or price sensitivity evaluation.

## Conclusion

The results from our study demonstrate that the MOC has high and broad appeal in all three countries. Feedback from the end users and HCPs was consistent across the countries for the various product attributes with only some differences. Product developers can use this information to modify and improve the final MOC design. There were some concerns among the study participants and HCPs regarding the potential for unpredictable side effects such as bleeding changes or, from the HCPs, regarding product safety and possible contraindications. The overall high appeal of the MOC was based on its value proposition for important contraceptive needs including convenient duration, ease of use, discretion, and acceptable side effects. Future clinical research on a hormonal MOC should include an exploration of women's tolerance and acceptability of the side effects, particularly regarding bleeding, to validate its value proposition.

## Data Availability

The raw data supporting the conclusions of this article will be made available by the authors, without undue reservation.
