# Corrigendum: Social and behavioral research with end-users and healthcare providers into understanding perceptions of and reactions to a monthly oral contraceptive capsule in Bangladesh, Senegal and Zimbabwe

**DOI:** 10.3389/fgwh.2025.1578982

**Published:** 2025-03-03

**Authors:** Moushira El-Sahn, Rose Elliott, Trisha Wood Santos, Mona El-Sahn, Jeff Lucas

**Affiliations:** ^1^ Routes2Results, Sevenoaks, United Kingdom; ^2^ Routes2Results, Aberdeen, United Kingdom; ^3^ Trisha Wood Santos Consulting, LLC, Seattle, WA, United States; ^4^ Routes2Results, London, United Kingdom

**Keywords:** family planning, acceptability, preferences, contraceptives in development, Africa, Asia, women, attributes

A Corrigendum on Social and behavioral research with end-users and healthcare providers into understanding perceptions of and reactions to a monthly oral contraceptive capsule in Bangladesh, Senegal and Zimbabwe El-Sahn M, Elliott R, Santos TW, El-Sahn M and Lucas J (2024). Front. Glob. Womens Health 5:1433189. doi: 10.3389/fgwh.2024.1433189

**Error in the Article Title**

In the published article, there was an error in the article title. Instead of “Understanding the perceptions and reactions to a monthly oral contraceptive capsule with end users and healthcare providers in Bangladesh, Senegal, and Zimbabwe”, it should be “Social and behavioral research with end-users and healthcare providers into understanding perceptions of and reactions to a monthly oral contraceptive capsule in Bangladesh, Senegal and Zimbabwe”.

**Error in Author List**

In the published article, there was an error in the order of the author list. The corrected author list appears below.

Moushira El-Sahn

Rose Elliott

Trisha Wood Santos

Mona El-Sahn

Jeff Lucas

The authors apologize for this error and state that this does not change the scientific conclusions of the article in any way. The original article has been updated.

