# Supplementary material for: Social and behavioral research with end-users and healthcare providers into understanding perceptions of and reactions to a monthly oral contraceptive capsule in Bangladesh, Senegal and Zimbabwe
Source: Front Glob Womens Health. 2024 Dec 11;5:1433189. doi: 10.3389/fgwh.2024.1433189 (PMC11668765; doi:10.3389/fgwh.2024.1433189)

Supplementary Material

**Social and behavioral research with end-users and healthcare providers (HCPs) in Bangladesh, Senegal and Zimbabwe, understanding perceptions of and reactions to a monthly oral contraceptive capsule.**

Moushira El-Sahn*, Rose Elliott, Mona El-Sahn, Jeff Lucas , and Trisha Wood Santos

*** Correspondence:** Corresponding Author: Moushira El-Sahn [Moushira@routes2results.org](mailto:Moushira@routes2results.org)

**ABBREVIATIONS**

CAPI Computer Assisted Personal Interviewing

CTPP Consumer Target Product Profile

CHW Community Health Worker

FP Family Planning

HCP Healthcare Practitioner

HIV Human Immunodeficiency Virus

IDI In-Depth Interview

IUD Intrauterine Device

MOC Monthly Oral Contraception

MPT Multipurpose Prevention Technology

R2R Routes2Results

SEC Socio Economic Class

WRA Women of Reproductive Age

# Supplementary Figures and Tables

**Supplementary Figure 1. Capsule size and image stimuli**


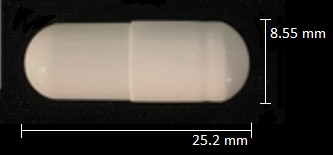

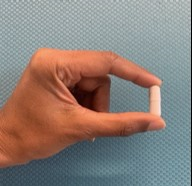


**Supplementary Figure 2. Consumer Target Product Profile (CTPP)**


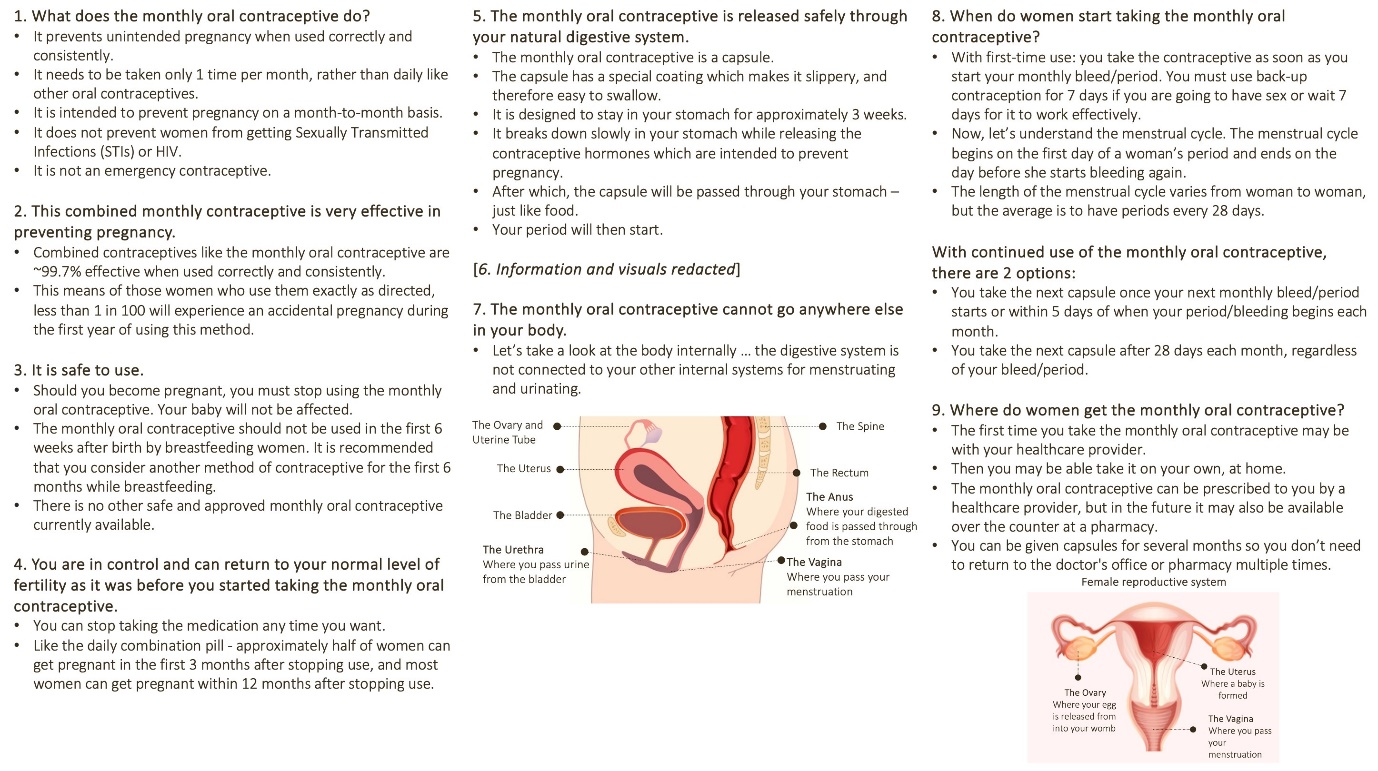


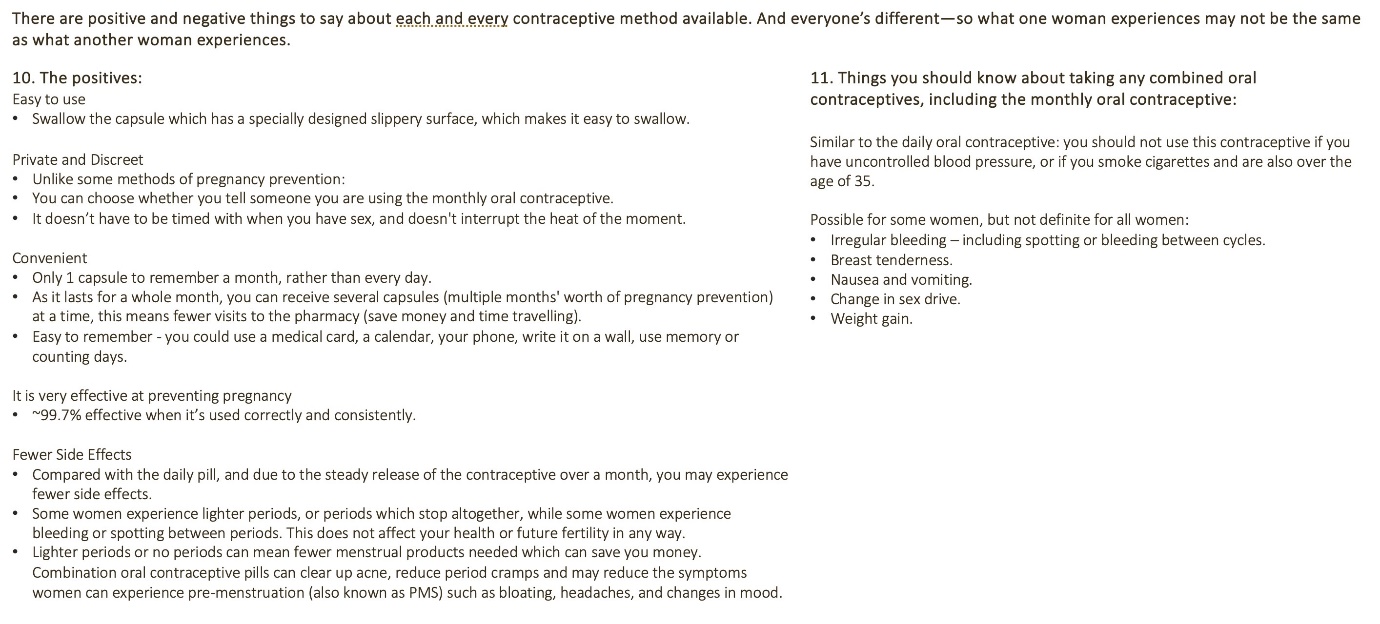

Supplement: Supplementary file 1 [file Table1.docx]
