# Supplementary material for: Social and behavioral research with end-users and healthcare providers into understanding perceptions of and reactions to a monthly oral contraceptive capsule in Bangladesh, Senegal and Zimbabwe
Source: Front Glob Womens Health. 2024 Dec 11;5:1433189. doi: 10.3389/fgwh.2024.1433189 (PMC11668765; doi:10.3389/fgwh.2024.1433189)
Supplement: Supplementary file 2 [file Table2.docx]

Figure captions

Table 1. Demographic and motherhood-related data, self-reported.

|  | Total  (n=1,825) | BGD  (n=617) | SEN  (n=601) | ZWE  (n=607) |  | Total  (n=1,825) | BGD  (n=617) | SEN  (n=601) | ZWE  (n=607) |
| --- | --- | --- | --- | --- | --- | --- | --- | --- | --- |
| Age (%) | | | | | Number of children (%) | | | | |
| 18-23 years old | 19.6 | 22.9 | 14.5↡ | 21.3 | None | 11.2 | 9.6 | 17.5↟ | 6.8 |
| 24-30 years old | 38.6 | 44.2 | 38.8 | 32.6↡ | 1 | 25.1 | 28.7 | 20.8 | 25.7 |
| 31-35 years old | 22.7 | 21.1 | 20.5 | 26.7↟ | 2 | 33.2 | 44.2↟ | 25.0 | 30.0 |
| 36-48 years old | 19.1 | 11.8 | 26.3↟ | 19.4 | 3 | 16.9 | 14.3 | 14.1 | 22.2↟ |
| Mean (years) | 29.87 | 28.49 | 31.10 | 30.04 | 4 | 7.8 | 2.8↡ | 11.5 | 9.4 |
| Relationship status (%) | | | | | 5 | 4.1 | 0.2↡ | 3.7 | 1.2 |
| Married live with* | 77.5 | 96.4↟ | 71.0 | 64.7 | 6 or more | 1.7 | 0.3↡ | 3.7 | 4.8 |
| Single + BFs*** | 9.7 | - | 16.1 | 13.2 | Mean | 1.95 | 1.75↡ | 2.05 | 2.06 |
| Married not live** | 6.3 | 3.4 | 7.5 | 8.1 | Education level (%) | | | | |
| Not living together | 1.8 | - | 0.2 | 5.3↟ | Primary + below | 19.6 | 26.7 | 22.5 | 9.6 |
| Living together | 1.6 | 0.2 | 0.7 | 4.1↟ | Junior secondary | 35.3 | 39.9 | 18.8↡ | 47.1↟ |
| Divorced | 1.5 | - | 2.5 | 2.1 | Senior secondary | 22.9 | 23.0↡ | 15.8 | 29.8 |
| Single no BF(s)*** | 1.1 | - | 1.7 | 1.6 | Higher education | 15.1 | 9.1 | 23.3↟ | 13.0 |
| Widowed | 0.4 | - | 0.3 | 0.8 |  | | | | |
| Country names using Alpha-3 codes Bangladesh (BGD), Senegal (SEN) and Zimbabwe (ZWE)  ↡significantly lower than the 2 other countries  ↟significantly higher than the 2 other countries  *Married living with partner  **Married not living with partner  ***Boy Friend(s) | | | | | | | | | |

Table 2. Distribution of HCP respondent specialties across countries (number of respondents, n=631).

|  | Total  (n=96) | BGD  (n=32) | SEN  (n=32) | ZWE  (n=32) | Total  (n=631) | BGD  (n=212) | SEN  (n=213) | ZWE  (n=206) |
| --- | --- | --- | --- | --- | --- | --- | --- | --- |
| (n=) | Qualitative component | | | | Quantitative component | | | |
| Doctor | 24 | 8 | 8 | 8 | 151 | 51 | 50 | 50 |
| Nurse/Family Welfare Visitors (FWV) or counsellors | 24 | 8 | 8 | 8 | 167 | 53 | 59 | 55 |
| Community Health Worker (CHW) | 24 | 8 | 8 | 8 | 155 | 52 | 52 | 51 |
| Pharmacist/medicine shop/medicine seller | 24 | 8 | 8 | 8 | 158 | 56 | 52 | 50 |
| Country names using Alpha-3 codes Bangladesh (BGD), Senegal (SEN) and Zimbabwe (ZWE) | | | | | | | | |

Table 3. End-user respondents’ unaided recollection of contraceptive methods.

|  | **Total**  (n=1,825) | **BGD**  (n=617) | **SEN**  (n=601) | **ZWE**  (n=607) |
| --- | --- | --- | --- | --- |
| **(%)** | | | | |
| Male condom | 47.9 | 74.4↟ | 18.0 | 50.7 |
| Oral birth control pill/'Daily pill' | 89.8 | 96.9↟ | 83.7 | 88.5 |
| Contraceptive injection/DMPA*/'Shot'/'Jab' | 57.0 | 72.0 | 73.4 | 25.7 |
| Oral emergency contraceptive pill/'Emergency pill' | 4.4 | 1.6 | 3.8 | 7.7 |
| Levonorgestrel Intrauterine system (LNG-IUS) or 'Hormonal Loop' | 18.6 | - | 4.3 | 51.7↟ |
| Intrauterine device (IUD)/Copper T ‘Non-hormonal Loop’ ‘Copper loop’ ‘Coil’ | 37.4 | 24.0 | 27.8 | 60.5↟ |
| Contraceptive implant/‘Rod’/‘Strand’ | 50.4 | 21.9 | 72.5↟ | 57.3 |
| Diaphragm/‘Cap’ | 0.8 | 1.0 | 0.5 | 1.0 |
| Female condom | 13.9 | - | 1.8 | 39.9↟ |
| Contraceptive Vaginal Ring (CVR) | 0.6 | - | 1.7 | 0.2 |
| Spermicide/spermicidal jelly, foam, suppository or film | 0.5 | - | 0.2 | 1.3 |
| Contraceptive patch | 0.2 | - | 0.5 | - |
| Counting days/Rhythm method | 1.5 | 0.2 | 3.8↟ | 0.5 |
| Withdraw | 0.8 | - | - | 2.5 |
| Other methods | 4.1 | 2.8 | 2.8 | 6.8↟ |
| None | 0.3 | 0.3 | 0.2 | 0.5 |
| Country names using Alpha-3 codes Bangladesh (BGD), Senegal (SEN) and Zimbabwe (ZWE)  ↡significantly lower than the 2 other countries  ↟significantly higher than the 2 other countries  *Depot medroxyprogesterone acetate | | | | |

Table 4. End-user respondents’ aided recollection of contraceptive methods.

|  | **Total** | **BGD** | **SEN** | **ZWE** |
| --- | --- | --- | --- | --- |
| **Summary of top score (%)** | | | | |
| Male condom | 77.4 | 93.5 | 45.6 | 92.4 |
| Oral contraceptive pill | 94.9 | 96.8 | 91.7 | 96.2 |
| DMPA | 87.1 | 90.4↟ | 85.0 | 85.7 |
| Oral emergency contraceptive pill | 39.2 | 36.3 | 35.4 | 46.0↟ |
| LNG-IUS | 26.2 | 5.2 | 26.5 | 47.3↟ |
| IUD | 48.5 | 45.9 | 39.1 | 60.5↟ |
| Contraceptive implant | 69.3 | 48.8 | 77.5 | 82.0 |
| DMPA-SC*/Bubble injection/Sayana Press | 5.2 | 4.4 | 9.7↟ | 1.5 |
| Diaphragm | 7.1 | 4.4 | 5.3 | 11.7 |
| Female condom | 38.8 | 8.8 | 22.1 | 86.0 |
| Contraceptive vaginal ring (CVR) | 7.5 | 6.8 | 8.3 | 7.2 |
| Spermicide | 4.6 | 3.1 | 3.0 | 7.7↟ |
| Contraceptive patch | 3.3 | 3.1 | 4.3 | 2.6 |
| Counting days/Rhythm method | 5.8 | 1.6 | 7.5 | 8.2 |
| Withdraw | 2.1 | - | - | 6.3 |
| Other methods | 0.9 | 0.3 | 1.3 | 1.2 |
| None | 0 | 0 | 0 | 0 |
| ↡significantly lower than the 2 other countries  ↟significantly higher than the 2 other countries  * Depot medroxyprogesterone acetate sub cutaneous (DMPA-SC). | | | | |

Table 5. Contraceptive methods ever used by end-user respondent.

|  | **Total**  n=1,820 | **BGD**  n=606 | **SEN**  n=598 | **ZWE**  n=616 |
| --- | --- | --- | --- | --- |
| **(%)** | | | | |
| Male condom | 52.2 | 70.5↟ | 23.7 | 61.7 |
| Oral contraceptive pill | 73.5 | 87.8↟ | 49.2 | 82.8 |
| Contraceptive injection | 36.5 | 37.3 | 37.1 | 35.0 |
| Oral emergency contraceptive pill | 13.1 | 13.3 | 10.5 | 15.5 |
| LNG-IUS | 1.3 | 0.2 | 2.2 | 1.7 |
| IUD | 4.9 | 3.1 | 7.0 | 4.6 |
| Contraceptive implant | 20.2 | 5.5 | 31.1↟ | 24.4 |
| DMPA-SC | 0.8 | - | 2.5 | - |
| Diaphragm | 0.4 | - | 0.8 | 0.5 |
| Female condom | 6.0 | 1.1 | 4.2 | 12.7↟ |
| CVR | 0.7 | 0.3 | 1.5↟ | 0.3 |
| Spermicide | 0.3 | - | 0.5 | 0.3 |
| Contraceptive patch | 0.2 | - | < 0.1 | - |
| Other methods* | 3.1 | 1.0 | 6.7↟ | 1.8 |
| None | 3.1 | 1.0 | 6.7 | 1.8 |
| Country names using Alpha-3 codes Bangladesh (BGD), Senegal (SEN) and Zimbabwe (ZWE)  ↡significantly lower than the 2 other countries  ↟significantly higher than the 2 other countries  *Such as natural family planning, “the rhythm method,”/ “Standard/Calendar days” / ‘Counting beads’ method | | | | |

Table 6. Contraceptives currently being used by end-user respondents.

|  | **Total**  (n=1,584) | **BGD**  (n=545) | **SEN**  (n=498) | **ZWE**  (n=541) |
| --- | --- | --- | --- | --- |
| **(%)** | | | | |
| Male condom | 11.0 | 19.4↟ | 5.6 | 7.6 |
| Oral contraceptive pill | 48.2 | 58.7 | 30.3↡ | 54.0 |
| Contraceptive injection | 16.7 | 14.7 | 23.3↟ | 12.8 |
| Oral emergency contraceptive pill | 1.2 | 1.5 | 1.0 | 1.1 |
| LNG-IUS | 0.8 | - | 1.6 | 0.9 |
| IUD | 2.7 | 0.4 | 5.2↟ | 2.8 |
| Contraceptive implant | 13.9 | 1.8 | 24.7↟ | 16.1 |
| DMPA-SC | 0.5 | - | 1.6 | - |
| Diaphragm | 0.3 | - | 0.6 | 0.2 |
| Female condom | 0.7 | 0.4 | 0.6 | 1.1 |
| Contraceptive vaginal ring (CVR) | 0.3 | - | 1.0 | - |
| Contraceptive patch | 0.1 | - | 0.4 | - |
| Other methods | 0.9 | 0.7 | 1.6 | 0.4 |
| None | 11.1 | 9.2 | 14.1 | 10.4 |
| Mean | 1.04 | 1.03 | 1.05 | 1.06 |
| Country names using Alpha-3 codes Bangladesh (BGD), Senegal (SEN) and Zimbabwe (ZWE)  ↡significantly lower than the 2 other countries  ↟significantly higher than the 2 other countries  *Such as natural family planning, “the rhythm method,”/ “Standard/Calendar days” / ‘Counting beads’ method | | | | |

Table 7. Prescription/hormonal contraceptives ever provided (or recommended in case of non-prescribers) by HCP respondents.

|  | **Total**  (n=631) | **BGD**  (n=212) | | **SEN**  (n=213) | **ZWE**  (n=206) |
| --- | --- | --- | --- | --- | --- |
| **Contraceptive methods ever provided (%)** | | | | | |
| Contraceptive pill | 98.6 | | 99.5 | 99.1 | 97.1 |
| Contraceptive injection | 92.4 | | 89.6 | 99.1 | 88.3 |
| IUD | 64.3 | | 59.9 | 88.3 | 44.2↡ |
| Contraceptive implant | 76.1 | | 62.3 | 88.7 | 77.2 |
| LNG-IUS | 21.7 | | 4.7↡ | 39.9↟ | 20.4 |
| Contraceptive vaginal ring (CVR) | 10.6 | | 2.8↡ | 22.1 | 6.8 |
| Another form of prescription contraception | 21.4 | | 14.2 | 15.0 | 35.4 |
| Country names using Alpha-3 codes Bangladesh (BGD), Senegal (SEN) and Zimbabwe (ZWE)  ↡significantly lower than the 2 other countries  ↟significantly higher than the 2 other countries | | | | | |

Table 8. Prescription/hormonal contraceptives provided (or recommended in case of non-prescribers) in the 3 months preceding the study by HCP respondents.

|  | **Total**  (n=631) | **BGD**  (n=212) | **SEN**  (n=213) | **ZWE**  (n=206) |
| --- | --- | --- | --- | --- |
| **Contraceptive methods provided in the last 3 months, summary of means** (%) | | | | |
| Contraceptive pill | 44.2 | 53.6 | 23.5↡ | 56.2 |
| Contraceptive injection | 25.8 | 30.7 | 25.8 | 20.7 |
| IUD | 16.8 | 14.0 | 20.8 | 12.4 |
| Contraceptive implant | 21.6 | 13.5 | 29.5↟ | 18.9 |
| LNG-IUS | 10.6 | 3.7↡ | 11.8 | 9.8 |
| Contraceptive vaginal ring (CVR) | 6.9 | 7.5 | 6.2 | 9.0 |
| Another form of prescription contraception | 10.5 | 14.0 | 3.4↡ | 12.2 |
| Country names using Alpha-3 codes Bangladesh (BGD), Senegal (SEN) and Zimbabwe (ZWE)  ↡significantly lower than the 2 other countries  ↟significantly higher than the 2 other countries | | | | |

Table 9. HCP perceptions of women’s compliance with contraceptives’ instructions for use.

|  | **Total**  (n=631) | **BGD**  (n=212) | **SEN**  (n=213) | **ZWE**  (n=206) |
| --- | --- | --- | --- | --- |
| **Summary of means (%)** | | | | |
| Poor compliance | 14.5 | 6.1↡ | 23.3 | 14.0 |
| Moderate compliance | 22.4 | 11.1↡ | 28.9 | 27.3 |
| Good compliance | 52.8 | 75.4 | 32.6↡ | 50.5 |
| Unknown compliance | 10.3 | 7.4 | 15.2 | 8.2 |
| Country names using Alpha-3 codes Bangladesh (BGD), Senegal (SEN) and Zimbabwe (ZWE)  ↡significantly lower than the 2 other countries  ↟significantly higher than the 2 other countries | | | | |

Table 10. Importance of features of a contraceptive method for end-user respondents.

|  | **Total**  (n=1,824) | **BGD**  (n=617) | **SEN**  (n=601) | **ZWE**  (n=607) |
| --- | --- | --- | --- | --- |
| **Top 2 Box scores, 4 + 5 out of 5 (%)** | | | | |
| It does not cause side effects such as headaches or weight gain | 81.4 | 85.7 | 76.5 | 81.9 |
| When side effects occur, they are not severe enough to disrupt my daily life | 82.7 | 86.7 | 76.0 | 85.3 |
| It allows me to become pregnant within 6 months of discontinuing it | 82.3 | 93.5↟ | 74.7 | 78.4 |
| It is discreet. No one knows that I am taking/using it | 83.5 | 89.3 | 91.7 | 69.4 |
| It does not cause irregular periods while using it | 83.2 | 87.4 | 77.7 | 84.5 |
| It does not cause my periods to stop while using it | 82.0 | 87.5↟ | 79.0 | 79.2 |
| It does not have to be taken every day | 78.8 | 83.1 | 79.9 | 73.5 |
| It is easy to remember when to take it or renew it | 89.6 | 92.9 | 86.2 | 89.8 |
| It protects against disease/infection | 78.1 | 88.5↟ | 71.5 | 74.0 |
| Country names using Alpha-3 codes Bangladesh (BGD), Senegal (SEN) and Zimbabwe (ZWE)  ↡significantly lower than the 2 other countries  ↟significantly higher than the 2 other countries | | | | |

Table 11. How far end-user respondents agree that the MOC fulfils specified features of a contraceptive method.

|  | **Total**  (n=1,824) | **BGD**  (n=617) | **SEN**  (n=601) | **ZWE**  (n=607) |
| --- | --- | --- | --- | --- |
| **Top 2 Box scores, 4 + 5 out of 5 (%)** | | | | |
| It may reduce period cramps and may reduce the symptoms women can experience pre-menstruation (PMS) such as bloating, headaches, and changes in mood | 60.5 | 67.7↟ | 55.2 | 58.3 |
| It will allow me to become pregnant within 6 months of discontinuing it | 71.1 | 84.4↟ | 58.4 | 70.2 |
| It is discreet. No one knows that I am taking/using it | 86.1 | 88.0 | 83.4 | 87.0 |
| It may clear up acne | 52.3 | 61.8↟ | 50.2 | 44.8 |
| It may cause my periods to stop while using it | 37.0 | 40.0 | 33.6 | 37.2 |
| It may cause spotting or bleeding between periods | 39.0 | 46.8↟ | 31.6 | 38.4 |
| It is easy to remember when to take it or renew it | 88.7 | 95.5↟ | 81.4 | 89.1 |
| It may cause my period to occur less than once a month | 49.3 | 51.4 | 46.9 | 49.4 |
| Country names using Alpha-3 codes Bangladesh (BGD), Senegal (SEN) and Zimbabwe (ZWE)  ↡significantly lower than the 2 other countries  ↟significantly higher than the 2 other countries | | | | |

Table 12. End-user respondents reasons for discontinuing the oral contraceptive pill.

|  | **Total**  (n=606) | **BGD**  (n=235) | **SEN**  (n=156) | **ZWE**  (n=215) |
| --- | --- | --- | --- | --- |
| **(%)** | | | | |
| It was hard to remember to take it every day/not convenient | 33.3 | 22.1↡ | 39.1 | 41.4 |
| I experienced headaches/migraines from it | 24.8 | 40.0↟ | 12.2 | 17.2 |
| It upset my stomach/caused nausea | 22.4 | 43.4↟ | 5.8 | 11.6 |
| I gained weight from it | 16.7 | 21.3 | 16.0 | 12.1 |
| I didn’t like it | 15.2 | 16.6 | 12.8 | 15.3 |
| I didn’t like that my periods became irregular while I was using it | 8.3 | 4.7 | 7.1 | 13.0 |
| It wasn’t very effective at preventing pregnancy | 4.8 | 2.6 | 3.8 | 7.9 |
| It was difficult to find/not available | 4.5 | 0.4 | <0.1 | 12.1 |
| I haven’t needed contraception since I stopped taking the contraception pill | 4.1 | 0.4 | 6.4 | 6.5 |
| My friends suggested I try something different | 3.8 | 1.3 | 1.3 | 8.4 |
| My doctor/nurse suggested I try something different | 3.6 | 2.6 | 5.1 | 3.7 |
| My partner did not like it | 3.5 | 2.6 | 4.5 | 3.7 |
| I had difficulty swallowing it | 3.1 | 5.5 | <0.1 | 2.8 |
| I heard bad/worrying things about it | 3.0 | 2.1 | 2.6 | 4.2 |
| I was worried it may prevent me from being able to have a baby in the future if I decide I want one/another one | 2.8 | 2.6 | 5.8 | 0.9 |
| I had to travel long distances to get it | 2.1 | 0.9 | <0.1 | 5.1 |
| I didn’t like that it reduced my monthly bleeding | 1.7 | 2.1 | 0.6 | 1.9 |
| I didn’t like that it stopped my monthly bleeding | 1.7 | 0.9 | 3.8 | 0.9 |
| It was too expensive | 1.5 | 1.3 | 0.6 | 2.3 |
| I didn’t like still getting my periods while using it | 1.5 | 1.3 | 0.6 | 2.3 |
| It wasn’t very effective at stopping the spread of disease/infections | 1.0 | - | 1.9 | 1.4 |
| Country names using Alpha-3 codes Bangladesh (BGD), Senegal (SEN) and Zimbabwe (ZWE)  ↡significantly lower than the 2 other countries  ↟significantly higher than the 2 other countries | | | | |

Table 13. Unscheduled bleeding patterns experienced by end-user respondents while using a contraceptive.

|  | **Total**  (n=1,763) | **BGD**  (n=610) | **SEN**  (n=558) | **ZWE**  (n=595) |
| --- | --- | --- | --- | --- |
| **(%)** | | | | |
| Spotting in between periods | 26.7 | 19.6 | 26.2 | 34.4↟ |
| Prolonged bleeding/bleeding for longer than usual | 21.9 | 17.4 | 17.4 | 30.7↟ |
| Bleeding occurring less frequently than once a month | 32.1 | 43.3↟ | 24.7 | 26.1 |
| Bleeding occurring more frequently than once a month | 23.6 | 22.2 | 18.6 | 29.6↟ |
| None of the above | 42.6 | 41.2 | 47.0 | 40.0 |
| Country names using Alpha-3 codes Bangladesh (BGD), Senegal (SEN) and Zimbabwe (ZWE)  ↡significantly lower than the 2 other countries  ↟significantly higher than the 2 other countries | | | | |

Table 14. Levels of concern felt by end-users due to experiencing unscheduled bleeding patterns while using a contraceptive.

|  | **Total**  (n=1,204) | **BGD**  (n=396) | **SEN**  (n=391) | **ZWE**  (n=417) |
| --- | --- | --- | --- | --- |
| **(%) and mean score** | | | | |
| **Spotting in between periods** (n=) | 470 | 120 | 143 | 207 |
| Very concerned (3) | 41.3 | 55.0 | 15.4↡ | 51.2 |
| Somewhat concerned (2) | 29.6 | 31.7 | 37.1 | 23.2 |
| Not at all concerned (1) | 25.1 | 12.5 | 39.9↟ | 22.2 |
| I don’t remember | 4.0 | 0.8 | 7.7 | 3.4 |
| Mean | 2.17 | 2.43 | 1.73↡ | 2.30 |
| **Prolonged bleeding/bleeding for longer than usual** (n=) | 393 | 109 | 99 | 185 |
| Very concerned (3) | 65.1 | 59.6 | 52.5 | 75.1↟ |
| Somewhat concerned (2) | 17.3 | 22.9 | 23.2 | 10.8 |
| Not at all concerned (1) | 13.2 | 16.5 | 14.1 | 10.8 |
| I don’t remember | 4.3 | 0.9 | 10.1 | 3.2 |
| Mean | 2.54 | 2.44 | 2.43 | 2.66↟ |
| **Bleeding occurring less frequently than once a month** (n=) | 566 | 270 | 139 | 157 |
| Very concerned (3) | 37.8 | 38.1 | 27.3↡ | 46.5 |
| Somewhat concerned (2) | 31.8 | 40.0 | 33.8 | 15.9 |
| Not at all concerned (1) | 25.6 | 19.6↡ | 33.1 | 29.3 |
| I don’t remember | 4.8 | 2.2 | 5.8 | 8.3 |
| Mean | 2.13 | 2.19 | 1.94↡ | 2.19 |
| **Bleeding occurring more frequently than once a month** (n=) | 423 | 137 | 110 | 176 |
| Very concerned (3) | 61.0 | 58.4 | 40.0↡ | 76.1 |
| Somewhat concerned (2) | 19.1 | 25.5 | 28.2 | 8.5↡ |
| Not at all concerned (1) | 13.5 | 13.9 | 18.2 | 10.2 |
| I don’t remember | 6.4 | 2.2 | 13.6 | 5.1 |
| Mean | 2.51 | 2.46 | 2.25↡ | 2.69 |
| **Period completely stopping** (n=) | 550 | 134 | 222 | 194 |
| Very concerned (3) | 53.6 | 72.4↟ | 43.2 | 52.6 |
| Somewhat concerned (2) | 16.5 | 10.4 | 25.2↟ | 10.8 |
| Not at all concerned (1) | 23.8 | 15.7 | 23.4 | 29.9 |
| I don’t remember | 6.0 | 1.5 | 8.1 | 6.7 |
| Mean | 2.32 | 2.58 | 2.22 | 2.24 |
| Country names using Alpha-3 codes Bangladesh (BGD), Senegal (SEN) and Zimbabwe (ZWE)  ↡significantly lower than the 2 other countries  ↟significantly higher than the 2 other countries | | | | |

Table 15. Menstrual side-effects about which women were reported to express concern either very frequently or frequently by HCP respondents.

|  | **Total**  (n=631) | **BGD**  (n=212) | **SEN**  (n=213) | **ZWE**  (n=206) |
| --- | --- | --- | --- | --- |
| **Top 2 Box scores, 4 + 5 out of 5 (%)** | | | | |
| Causing episodic bleeding or spotting | 77.7 | 76.9 | 76.1 | 80.1 |
| Causing missed menses | 76.1 | 71.7 | 85.9↟ | 70.4 |
| Causing amenorrhea (no bleeding at all) | 61.6 | 55.7 | 76.1↟ | 52.9 |
| Causing prolonged bleeding | 65.5 | 67.0 | 53.5↡ | 76.2 |
| Country names using Alpha-3 codes Bangladesh (BGD), Senegal (SEN) and Zimbabwe (ZWE)  ↡significantly lower than the 2 other countries  ↟significantly higher than the 2 other countries | | | | |
